# Muscarinic Receptors Are Responsible for the Cholinergic Modulation of Projection Neurons in the Song Production Brain Nucleus RA of Zebra Finches

**DOI:** 10.3389/fncel.2017.00051

**Published:** 2017-02-28

**Authors:** Wei Meng, Songhua Wang, Lihua Yao, Nan Zhang, Dongfeng Li

**Affiliations:** ^1^Jiangxi Key Laboratory of Organic Chemistry, Jiangxi Science and Technology Normal UniversityNanchang, China; ^2^School of Life Science, South China Normal UniversityGuangzhou, China; ^3^School of Life Science, Jiangxi Science and Technology Normal UniversityNanchang, China

**Keywords:** cholinergic modulation, mAChR, nAChR, projection neuron, song premotor nucleus, RA, zebra finch

## Abstract

Songbirds are a useful model for the study of learned vocal behavior in vertebrates. The robust nucleus of the arcopallium (RA) is a premotor nucleus in the vocal motor pathway. It receives excitatory synaptic inputs from the anterior forebrain pathway. RA also receives cholinergic inputs from the ventral paleostriatum of the basal forebrain. Our previous study showed that carbachol, a non-selective cholinergic receptor agonist, modulates the electrophysiology of RA projection neurons (PNs), indicating that cholinergic modulation of RA may play an important role in song production. However, the receptor mechanisms underlying these effects are poorly understood. In the present study, we investigated the electrophysiological properties of two acetylcholine receptors on the RA PNs of adult male zebra finches using *in vitro* whole-cell current clamp. Our results demonstrate that activation of muscarinic acetylcholine receptors (mAChRs) simulate the effects of carbachol. Both carbachol and the mAChR agonist muscarine produced a decrease in the excitability of RA PNs and a hyperpolarization of the membrane potential. The mAChR antagonist atropine blocked the effects of carbachol. Activation of nicotinic acetylcholine receptors (nAChRs) with nAChR agonist nicotine or DMPP had no effect on the excitability of RA PNs, and the nAChR antagonist mecamylamine failed to inhibit the effects of carbachol. These results suggest that mAChRs, but not nAChRs, primarily modulate the effects of carbachol on the activity of RA PNs. Collectively, these findings contribute to our understanding of the mechanism of cholinergic modulation in the vocal nuclei of songbirds.

## Introduction

Songbirds represent one of the best animal models to investigate the role of neurotransmitters in learned vocal behavior. The song control system in the songbird’s brain comprises a network of inter-connected nuclei ranging from forebrain to brainstem (Nottebohm et al., 1976). In the forebrain of songbird, the vocal motor pathway (VMP) is required for song production, and the anterior forebrain pathway (AFP) controls the maintenance, learning and plasticity of song production (Foster and Bottjer, 2001; Brainard and Doupe, 2002; Rouse and Ball, 2016). The robust nucleus of the arcopallium (RA) is a premotor nucleus in VMP, receiving excitatory glutamate inputs from both the nucleus HVC (proper name) and the lateral magnocellular nucleus of the anterior nidopallium (LMAN) in AFP. The projection neurons (PNs) in RA project to the hypoglossal motor nucleus that innervates the muscles of syrinx (Vicario, 1994; Jarvis, 2004; Pfenning et al., 2014). RA exhibits electrophysiological activity that matches the song output (Yu and Margoliash, 1996; Chi and Margoliash, 2001). Moreover, the RA activity is associated with fluctuation in the entropy, pitch and amplitude of syllables (Sober et al., 2008). Lesions in RA lead to serious song deficits (Nottebohm et al., 1976). These findings demonstrate that RA plays a crucial role in birdsong production.

Acetylcholine (ACh) is an important neurotransmitter in vertebrates. In mammals, cholinergic inputs potentially regulate learning and plasticity of motor cortex (Conner et al., 2010). Songbird RA is functionally similar to motor cortex, and more specifically resembles laryngeal motor cortex (Pfenning et al., 2014). In songbirds, RA receives cholinergic innervations from the ventral paleostriatum (VP) in the basal forebrain (Li et al., 1999). Cholinergic fibers and neurons exist in RA (Sadananda, 2004). Both metabotropic muscarinic ACh receptors (mAChRs) and ionotropic nicotinic ACh receptors (nAChRs) exist in RA (Ryan and Arnold, 1981; Watson et al., 1988; Ball et al., 1990; Salgado-Commissariat et al., 2004).

Our previous study showed that carbachol, a non-selective cholinergic receptor agonist, reduces the excitability of RA PNs by hyperpolarizing membrane potential and increasing afterhyperpolarization (AHP) and membrane conductance (Meng et al., 2016), suggesting that cholinergic modulation of RA may play a critical role in song production. However, the receptor mechanisms underlying these effects are still unclear. In order to further understand the cholinergic modulation of RA, we investigated the electrophysiological effects of mAChRs and nAChRs on the RA PNs of adult male zebra finches using an *in vitro* whole-cell current clamp.

## Materials and Methods

### Animals and Brain Slice Preparation

Adult male zebra finches (*Taeniopygia guttata*; >120 days) were obtained from a supplier. The animal studies were approved by the Institutional Care and Use Committee of Jiangxi Science and Technology Normal University. The experimental methods were described in our previous studies (Wang et al., 2014, 2015; Meng et al., 2016). In brief, birds were anesthetized and euthanized by decapitation. The brains were dissected and immersed in ice cold, oxygenated (5% CO_2_ and 95% O_2_) solution containing (in mM) 62.5 NaCl, 5 KCl, 28 NaHCO_3_, 10 glucose, 1.3 MgSO_4_⋅7H_2_O, 1.26 NaH_2_PO_4_⋅H_2_O, and 248 sucrose (pH 7.4). Coronal brain slices measuring 250–300 μm in thickness and containing RA were obtained using a vibrating microtome (NVSLM1, World Precision Instruments, USA). Slices were collected at 37°C in oxygenated artificial cerebrospinal fluid (ACSF) containing (in mM) 25 glucose, 25 NaHCO_3_, 1.27 NaH_2_PO_4_⋅H_2_O, 2.5 KCl, 1.2 MgSO_4_⋅7H_2_O, 2.0 CaCl_2_, and 125 NaCl (pH 7.4). After 30 min, the slices in the holding chamber were allowed to recover at room temperature (22–26°C) for 1 h.

### Patch-Clamp Recording and Drug Application

After recovery, the slices were individually isolated in a recording chamber and superfused with oxygenated ACSF (2.0 mL/min) and room temperature. RA neurons were visualized under BX51WI microscope connected to a DIC-IR video camera (Olympus, Japan) at high magnification (40×). RA PNs were identified based on distinct electrophysiological properties as described previously (Spiro et al., 1999; Liao et al., 2011). The following experiments were performed using conventional whole-cell patch recordings under current-clamp configurations. The recording electrodes, fabricated from borosilicate glass pipettes (Sutter Instruments, USA), were pulled by a Flaming-Brown puller (P-97, Sutter Instruments, USA) and were filled with intracellular solution containing (in mM) 10 HEPES, 5 NaCl, 120 KMeSO_4_, 2 EGTA, 2 Mg-ATP, and 0.3 Na_3_-GTP (pH 7.3–7.4). The electrode resistance was 4–6 MΩ. RA PNs with a resting membrane potential greater (i.e., more positive) than -50 mV and series resistance (typically 10–20 MΩ) with changes >30% were abandoned.

The following pharmacological reagents were used: carbachol (30 μM); atropine sulfate (atropine, 10 μM); mecamylamine hydrochloride (mecamylamine, 10 μM); muscarine chloride hydrate (muscarine, 10 μM); (-)-nicotine hydrogen tartrate salt (nicotine, 10 μM) and 1,1-dimethyl-4-phenylpiperazinium iodide (DMPP, 10 μM). These drugs were obtained from Sigma-Aldrich. The effects of these drugs on RA PNs were tested by bath perfusion. Signals were amplified by MultiClamp 700B (Molecular Devices, USA). Signals were low-pass filtered at 5 kHz and digitized at 10 kHz with Digidata 1440A (Molecular Devices, USA).

### Data Analysis

The software pClamp 10.4 (Axon Instruments, USA) and Origin Pro 8.0 (Origin Lab, USA) were used for data acquisition and analysis. Electrophysiological properties were measured according to the procedures defined by Farries and Perkel (2000) and reported in our previous study (Meng et al., 2016). The membrane time constant was calculated by fitting a single exponential curve to the membrane potential change in response to -200 pA hyperpolarizing pulses. The membrane capacitance was calculated by dividing the membrane time constant by the membrane input resistance (Meitzen et al., 2009). The membrane input resistance was measured by a series of 600 ms hyperpolarizing current steps from -200 to 0 pA, step 20 pA with 10 s intervals. The slope of the current-voltage curve is designated as the membrane input resistance. All the data were presented as the means ± SEM and compared using paired two-tailed Student’s *t*-test (*p* < 0.05 shows statistical significance).

## Results

### mAChR Agonist Decreased the Evoked AP Firing of RA PNs

We used the mAChR agonist muscarine to determine its effects on the evoked AP firing and identify the type of ACh receptors mediating the regulation of RA PN activity.

**FIGURE 1:**
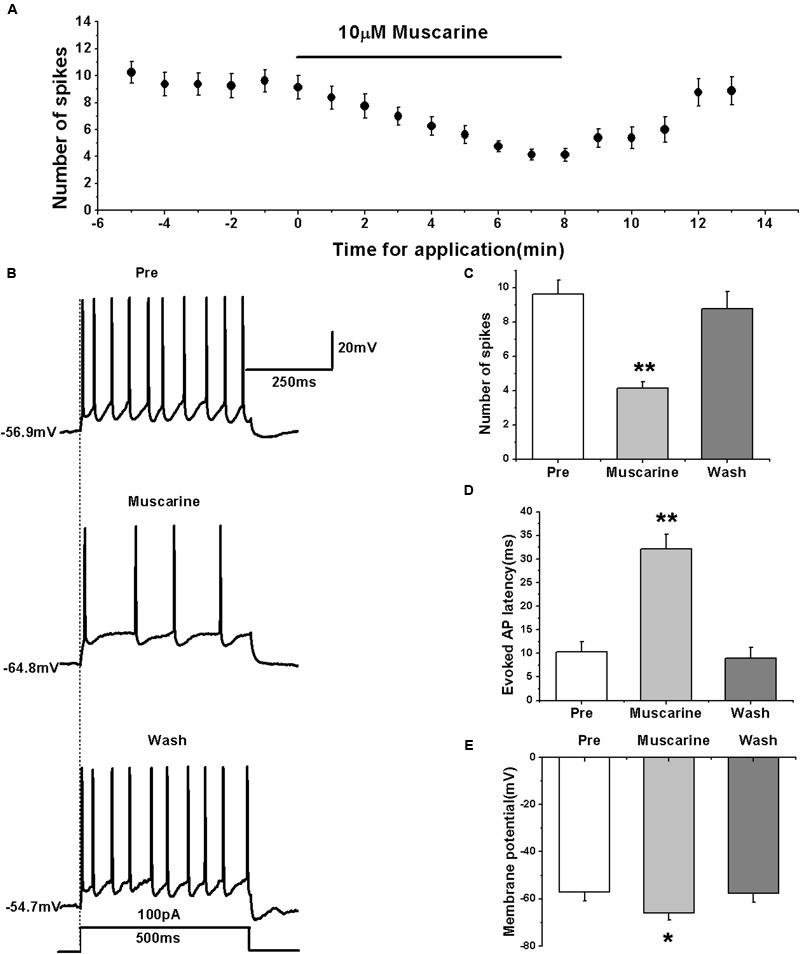
**Muscarine (10 μM) decreased the evoked AP firing of RA PNs. (A)** Time course of the number of evoked spikes in the presence of muscarine (*n* = 8). The line at the top indicates the drug present in the bath. **(B)** The traces illustrate the responses with consecutive treatment in a representative neuron. **(C)** The number of evoked spikes was significantly decreased in the presence of muscarine (*n* = 8). **(D)** Evoked AP latency was significantly increased in the presence of muscarine (*n* = 8). **(E)** Membrane potential was significantly hyperpolarized in the presence of muscarine (*n* = 8). ^∗^*p <* 0.05, ^∗∗^*p <* 0.01.

We tested the effects of muscarine by first evoking the AP firing of RA PNs with 100 pA of 500 ms duration and 1-min interval (**Figures 1A,B**). The results showed that muscarine significantly decreased the number of spikes from 9.63 ± 0.88 to 4.13 ± 0.43 (*n* = 8; *p* < 0.01; **Figure 1C**), and increased to 8.75 ± 1.08 (*n* = 8) after muscarine washout (**Figure 1C**). Moreover, muscarine markedly increased the evoked AP latency from 10.26 ± 2.40 to 32.16 ± 3.28 ms, recovering to 9.00 ± 2.35 ms after washout (**Figure 1D**), indicating that muscarine reduced the evoked AP firing of RA PNs. Meanwhile, muscarine induced the hyperpolarization of membrane potential from -57.17 ± 3.87 to -66.02 ± 3.06 mV (*n* = 8; *p* < 0.01), and recovery to -57.79 ± 3.77 mV occurred after muscarine washout (**Figure 1E**), indicating that the decrease in the excitation of RA PNs was triggered by muscarine via membrane hyperpolarization.

**FIGURE 2:**
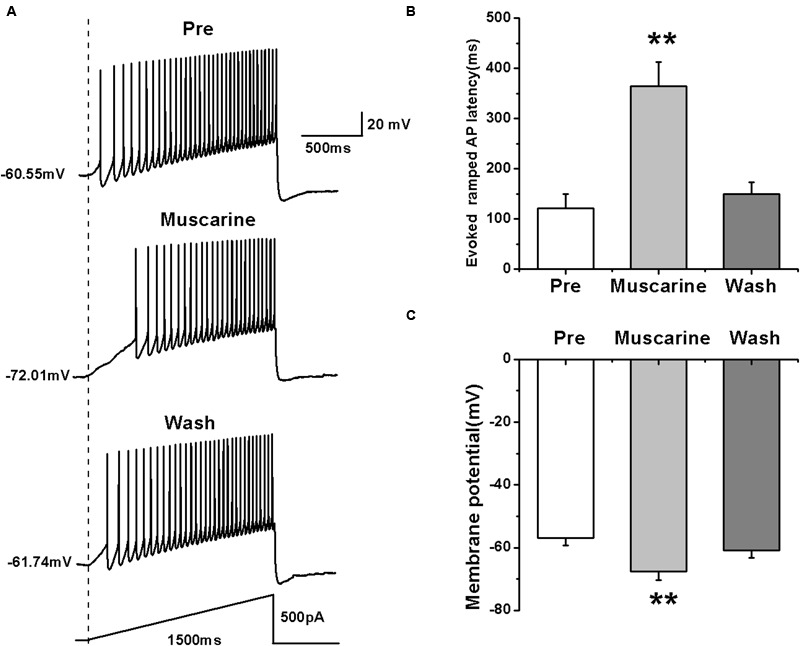
**Effects of muscarine (10 μM) on AP firing of RA PNs evoked by a depolarizing ramp stimulus, with a linear increase in intensity from 0 to 500 pA within 1500 ms. (A)** Sample traces of AP firing evoked by a ramp before, during and after muscarine application. **(B)** Evoked ramped AP latency was significantly increased in the presence of muscarine (*n* = 7). **(C)** Membrane potential was significantly hyperpolarized in the presence of muscarine (*n* = 7). ^∗∗^*p <* 0.01.

We further tested the effects of muscarine on the activity of RA PNs using another pattern of depolarizing stimulus — ramp, which increased in intensity from 0 to 500 pA linearly over 1500 ms (Yao et al., 2011; Meng et al., 2016) (**Figure 2A**). The results showed that muscarine increased the evoked ramped AP latency from 121.53 ± 27.71 to 364.74 ± 47.74 ms (*n* = 7; *p* < 0.01), which recovered to 149.89 ± 23.07 ms after muscarine washout (**Figure 2B**). In addition, muscarine also induced the hyperpolarization of the membrane potential from -56.99 ± 2.26 to -67.52 ± 2.91 mV (*n* = 7; *p* < 0.01; **Figure 2C**), which recovered to -60.96 ± 2.34 mV after muscarine washout (**Figure 2C**). These results further suggested that the muscarine-induced reduction of RA PN excitability may be due to membrane hyperpolarization.

### Effects of mAChR Agonist on the Intrinsic Properties of RA PNs

**FIGURE 3:**
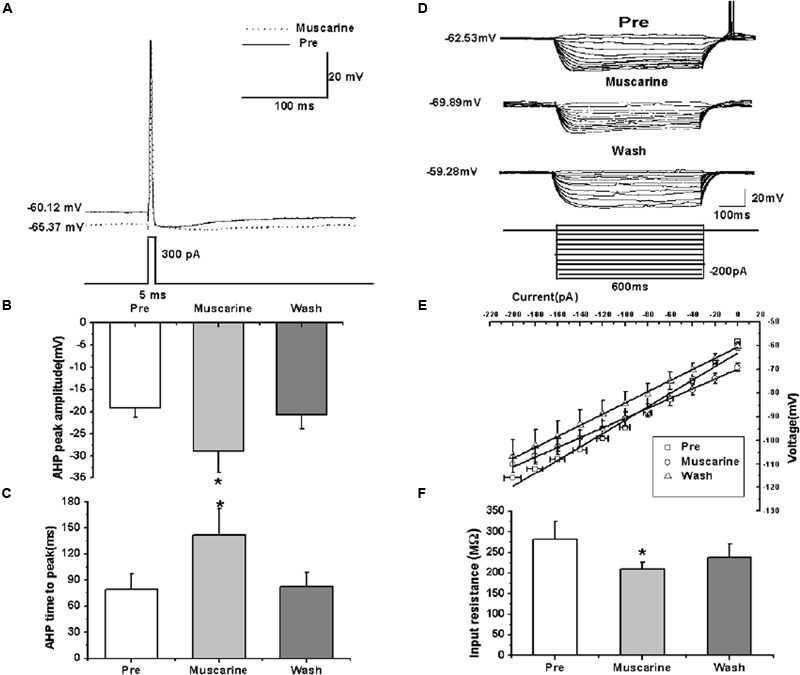
**Effects of muscarine (10 μM) on the intrinsic properties of RA PNs. (A)** Representative AP recordings in response to a depolarizing pulse of 300 pA at 5 ms duration before and during muscarine application. **(B)** AHP peak amplitude was significantly increased in the presence of muscarine (*n* = 8). **(C)** AHP time to peak was significantly increased in the presence of muscarine (*n* = 8). **(D)** Voltage responses of a neuron to a series of hyperpolarizing current steps before, during and after muscarine application. **(E)** The current-voltage curves showed a significant change in slope during muscarine application (*n* = 9), indicating muscarine effect on the membrane input resistance. **(F)** Membrane input resistance was significantly decreased in the presence of muscarine (*n* = 9). ^∗^*p* < 0.05.

We further analyzed the effects of muscarine on the AP of RA PNs evoked by a depolarizing current pulse of 300 pA at 5 ms (**Figure 3A**). The results showed a gradual recovery of the significantly hyperpolarized membrane potential (**Table 1**). It was accompanied with an increase in the AHP peak amplitude and AHP time to peak during muscarine application, and return to the control level following muscarine washout (**Table 1** and **Figures 3B,C**). However, the AP threshold, peak amplitude and half-width were unaffected (**Table 1**).

**Table 1:** Measurements of intrinsic properties of RA PNs before, during and after muscarine (10 μM) application.

Parameters	Pre	Muscarine	*t* values, *p* values	Wash
Membrane potential (mV, *n* = 8)	-60.12 ± 2.84	-66.13 ± 2.06**	*t* = 4.460, *p* = 0.003	-59.00 ± 2.77
AP threshold (mV, *n* = 8)	-43.61 ± 3.14	-37.69 ± 5.44	*t* = -2.078, *p* = 0.076	-40.63 ± 3.08
Peak amplitude (mV, *n* = 8)	63.95 ± 7.66	64.25 ± 7.56	*t* = -0.135, *p* = 0.915	63.33 ± 5.92
Half-width (ms, *n* = 8)	1.74 ± 0.15	1.61 ± 0.14	*t* = 1.330, *p* = 0.062	1.93 ± 0.31
AHP peak amplitude (mV, *n* = 8)	-19.10 ± 2.34	-28.88 ± 5.27*	*t* = 2.895, *p* = 0.023	-20.71 ± 3.33
AHP time to peak (ms, *n* = 8)	79.18 ± 19.40	141.91 ± 32.08*	*t* = -3.496, *p* = 0.010	82.63 ± 17.41
Membrane input resistance (MΩ, *n* = 9)	282.72 ± 42.23	208.98 ± 16.61*	*t* = 2.537, *p* = 0.035	237.44 ± 32.57
Membrane time constant (ms, *n* = 9)	27.93 ± 1.90	19.19 ± 1.39**	*t* = 3.850, *p* = 0.005	20.16 ± 2.45
Membrane capacitance (pF, *n* = 9)	109.44 ± 13.89	97.76 ± 12.32	*t* = 1.214, *p* = 0.259	88.53 ± 10.83

*^∗^*p <* 0.05, ^∗∗^*p <* 0.01.*

In addition, we also tested the roles of muscarine on membrane input resistance, membrane time constant and membrane capacitance of RA PNs. The results showed that the membrane input resistance was decreased during muscarine application, and recovered to the control level after muscarine washout (**Table 1** and **Figures 3D–F**). The membrane time constant was decreased as well during muscarine application, and recovered gradually after muscarine washout (**Table 1**). However, the membrane capacitance was unaffected (**Table 1**). Based on our previous study suggesting that carbachol also increased the AHP and membrane conductance of RA PNs (Meng et al., 2016), these results indicated that muscarine mimics the effects of carbachol on RA PNs.

### Effects of mAChR Antagonist on the Evoked AP Firing of RA PNs

**FIGURE 4:**
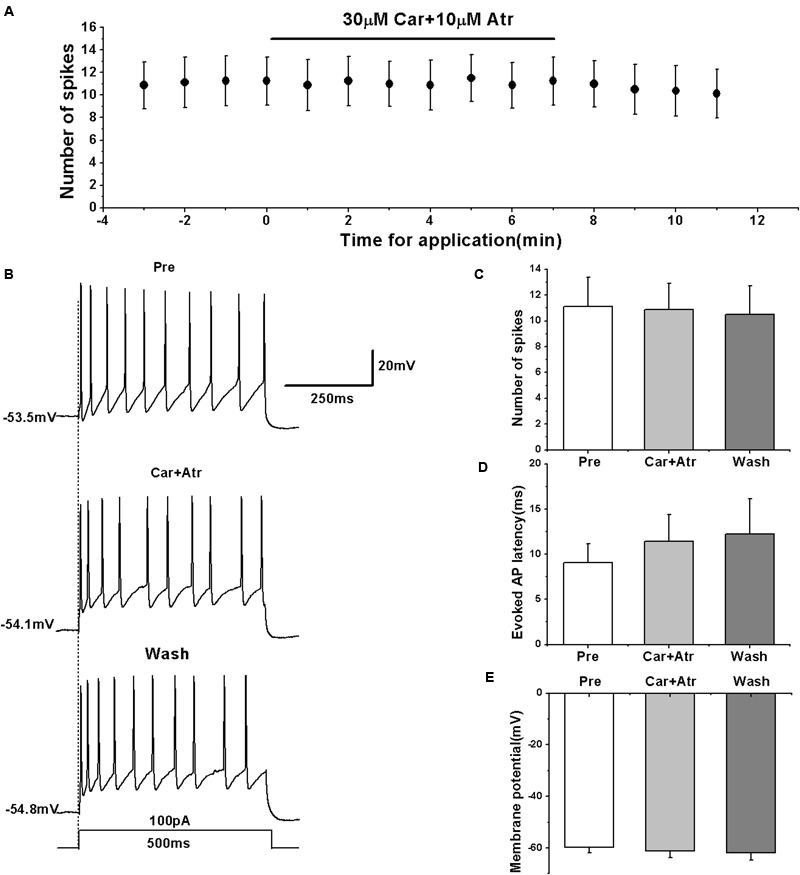
**Effects of carbachol (Car, 30 μM) + atropine (Atr, 10 μM) on the evoked AP firing of RA PNs. (A)** Time course of the number of evoked spikes in the presence of carbachol and atropine (*n* = 8). The line at the top indicates the drugs present in the bath. **(B)** The traces illustrate the responses with consecutive treatment in a representative neuron. **(C)** The number of evoked spikes was unchanged in the presence of Car + Atr (*n* = 8). **(D)** Evoked AP latency was unchanged in the presence of Car + Atr (*n* = 8). **(E)** Membrane potential was unchanged in the presence of Car + Atr (*n* = 8).

To further confirm the effects of mAChRs on the activity of RA PNs, we simultaneously tested the effects of carbachol and mAChR antagonist atropine on the evoked AP firing. We evoked the AP firing of RA PNs using a depolarizing stimulus of 100 pA at 500 ms duration and tested the effects of carbachol combined with atropine (**Figures 4A,B**). The results showed that carbachol had no effect on the number of evoked spikes in the presence of atropine (pre: 11.13 ± 2.41; carbachol + atropine: 10.88 ± 2.16; wash: 10.50 ± 2.36; *n* = 8; *p* > 0.05; **Figure 4C**). The combination of carbachol and atropine had no effect on the evoked AP latency (pre: 9.06 ± 2.21 ms; carbachol + atropine: 11.41 ± 3.15 ms; wash: 12.24 ± 4.15 ms; *n* = 8; *p* > 0.05; **Figure 4D**). The combination of carbachol and atropine also had no effect on the membrane potential (pre: -59.87 ± 2.25 mV; carbachol + atropine: -61.17 ± 2.91 mV; wash: -61.81 ± 3.14 mV; *n* = 8; *p* > 0.05; **Figure 4E**). These results indicate that mAChR antagonist blocked the effects of carbachol on RA PNs.

### nAChR Agonist Had No Effect on the Evoked AP Firing of RA PNs

**FIGURE 5:**
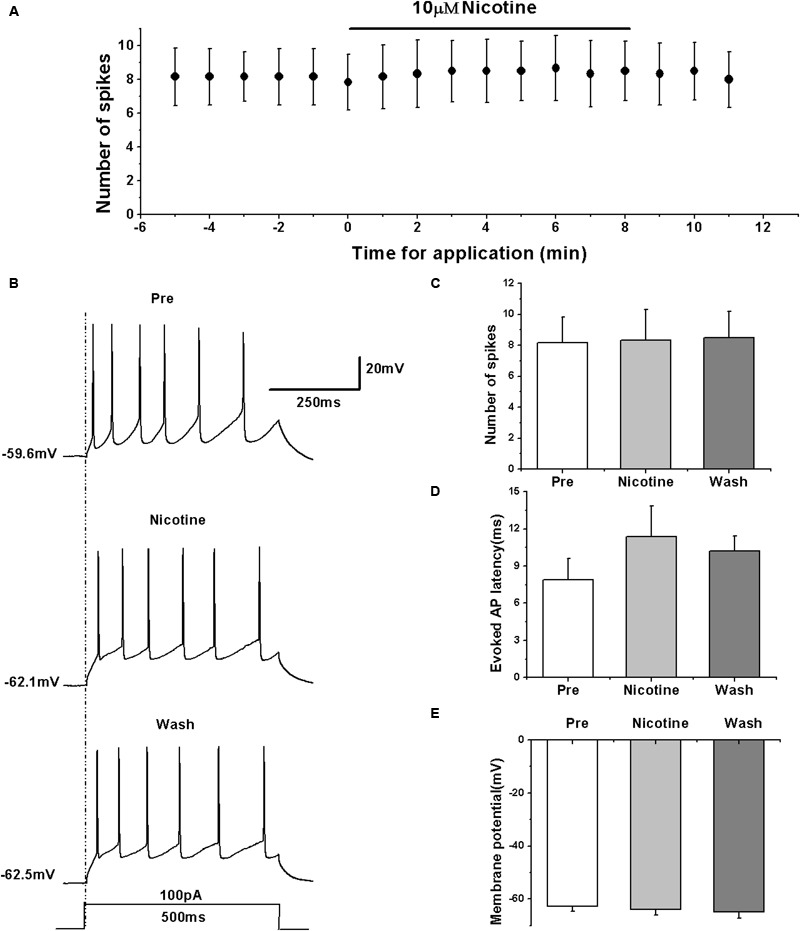
**Effects of nicotine (10 μM) on the evoked AP firing of RA PNs. (A)** Time course of the number of evoked spikes in the presence of nicotine (*n* = 6). The line at the top indicates the drug present in the bath. **(B)** The traces illustrate the responses with consecutive treatment in a representative neuron. **(C)** The number of evoked spikes was unchanged in the presence of nicotine (*n* = 6). **(D)** Evoked AP latency was unchanged in the presence of nicotine (*n* = 6). **(E)** Membrane potential was unchanged in the presence of nicotine (*n* = 6).

We determined the effects of nAChRs on the activity of RA PNs in terms of evoked AP using the nAChR agonist nicotine. We evoked the AP of RA PNs using a depolarizing current of 100 pA at 500 ms duration and tested the effects of nicotine (**Figures 5A,B**). The results showed that nicotine application has no effect on the number of evoked spikes (pre: 8.17 ± 1.82; nicotine: 8.33 ± 2.15; wash: 8.50 ± 1.87; *n* = 6; *p* > 0.05; **Figure 5C**) and the evoked AP latency (pre: 7.88 ± 1.90 ms; nicotine: 11.40 ± 2.71 ms; wash: 10.22 ± 1.33 ms; *n* = 6; *p* > 0.05; **Figure 5D**). Meanwhile, nicotine also had no effect on the membrane potential (pre: -62.82 ± 1.89 mV; nicotine: -63.94 ± 2.34 mV; wash: -64.74 ± 2.60 mV; *n* = 6; *p* > 0.05; **Figure 5E**). These results indicate that nAChRs may not regulate the activity of RA PNs.

**FIGURE 6:**
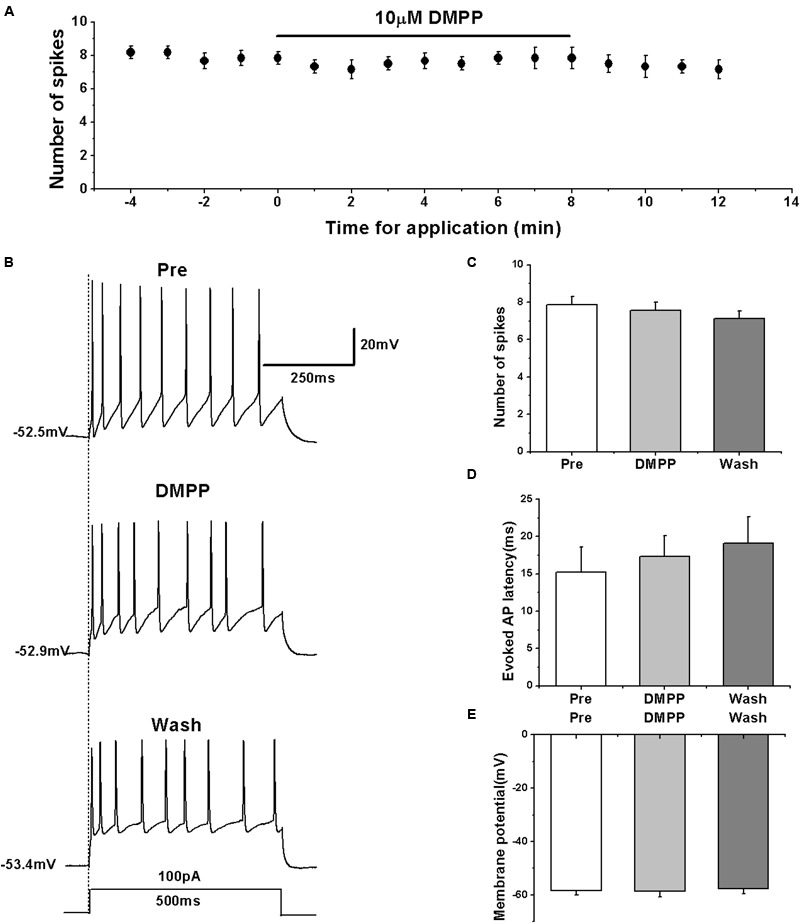
**Effects of DMPP (10 μM) on the evoked AP firing of RA PNs. (A)** Time course of the number of evoked spikes in the presence of DMPP (*n* = 7). The line at the top indicates the drug present in the bath. **(B)** The traces illustrate the responses with consecutive treatment in a representative neuron. **(C)** The number of evoked spikes was unchanged in the presence of DMPP (*n* = 7). **(D)** Evoked AP latency was unchanged in the presence of DMPP (*n* = 7). **(E)** Membrane potential was unchanged in the presence of DMPP (*n* = 7).

Previous studies showed that nicotine increased the frequency of evoked and spontaneous action potentials in RA neurons (Salgado-Commissariat et al., 2004), which is seemingly inconsistent with our results. However, the type of neurons recorded within RA was unclear in the study of Salgado-Commissariat et al. (2004). We further validated the effects of nAChRs on the activity of RA PNs using another nAChR agonist DMPP. The results showed that similar to the effects of nicotine, DMPP application had no effect on the number of evoked spikes (pre: 7.86 ± 0.50; DMPP: 7.57 ± 0.46; wash: 7.14 ± 0.44; *n* = 7; *p* > 0.05; **Figures 6A–C**) and the evoked AP latency (pre: 15.19 ± 3.63 ms; DMPP: 17.30 ± 3.06 ms; wash: 19.10 ± 3.83 ms; *n* = 7; *p* > 0.05; **Figure 6D**). DMPP also had no effect on the membrane potential (pre: -58.40 ± 1.83 mV; DMPP: -58.58 ± 2.24 mV; wash: -57.57 ± 2.27 mV; *n* = 7; *p* > 0.05; **Figure 6E**), further indicating that nAChRs do not regulate the activity of RA PNs.

### Effects of nAChR Antagonist on the Evoked AP Firing of RA PNs

To further clarify the effects of nAChRs on the activity of RA PNs, we simultaneously tested the effect of carbachol and nAChR antagonist mecamylamine on the evoked AP firing.

**FIGURE 7:**
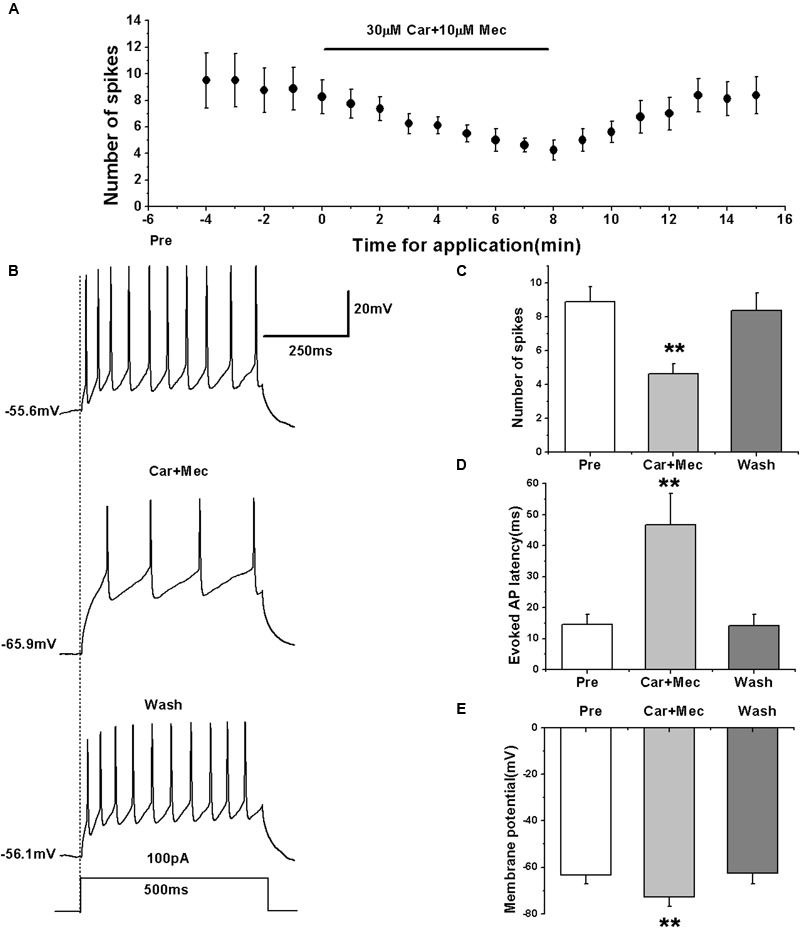
**Effects of carbachol (Car, 30 μM) + mecamylamine (Mec, 10 μM) on the evoked AP firing of RA PNs. (A)** Time course of the number of evoked spikes in the presence of carbachol and mecamylamine (*n* = 8). The line at the top indicates the drugs present in the bath. **(B)** The traces illustrate the responses with consecutive treatment in a representative neuron. **(C)** The number of evoked spikes was significantly decreased in the presence of Car + Mec (*n* = 8). **(D)** Evoked AP latency was significantly increased in the presence of Car + Mec (*n* = 8). **(E)** Membrane potential was significantly hyperpolarized in the presence of Car + Mec (*n* = 8). ^∗∗^*p <* 0.01.

We first evoked the AP firing of RA PNs with a depolarizing stimulus of 100 pA lasting 500 ms and tested the combined effects of carbachol and mecamylamine (**Figures 7A,B**). The results showed that similar to the effects of carbachol alone, the combination of carbachol and mecamylamine significantly decreased the number of evoked spikes (pre: 8.88 ± 0.98; carbachol + mecamylamine: 4.63 ± 0.64; wash: 8.38 ± 1.09; *n* = 8; *p* < 0.01; **Figure 7C**), increased the evoked AP latency (pre: 14.53 ± 3.56 ms; carbachol + mecamylamine: 46.69 ± 10.83 ms; wash: 14.18 ± 3.95 ms; *n* = 8; *p* < 0.01; **Figure 7D**), and induced the hyperpolarization of membrane potential (pre: -63.34 ± 4.09 mV; carbachol + mecamylamine: -72.67 ± 4.46 mV; wash: -62.58 ± 4.78 mV; *n* = 8; *p* < 0.01; **Figure 7E**).

**FIGURE 8:**
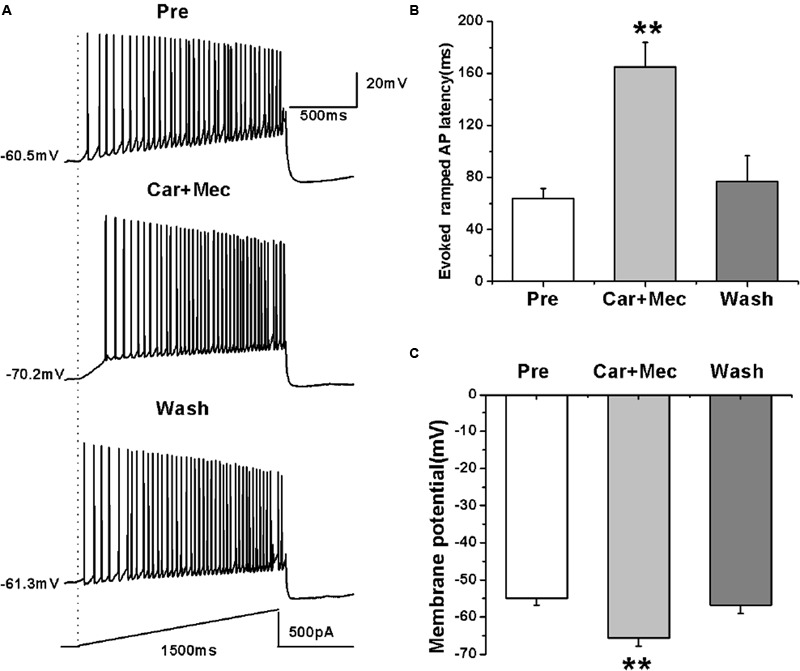
**Effects of carbachol (Car, 30 μM) + mecamylamine (Mec, 10 μM) on the AP firing of RA PNs evoked by a depolarizing ramp stimulus, with a linear increase in intensity from 0 to 500 pA within 1500 ms. (A)** Sample traces of AP firing evoked by a ramp before, during and after Car + Mec application. **(B)** Evoked ramped AP latency was significantly increased in the presence of Car + Mec (*n* = 7). **(C)** Membrane potential was significantly hyperpolarized in the presence of Car + Mec (*n* = 7). ^∗∗^*p* < 0.01.

We further tested the effects of carbachol plus mecamylamine on the evoked activity of RA PNs using a ramp (**Figure 8A**). Similar to the effects of carbachol alone, the use of carbachol combined with mecamylamine significantly increased the evoked ramped AP latency (pre: 64.08 ± 7.36 ms; carbachol + mecamylamine: 165.23 ± 18.87 ms; wash: 76.80 ± 19.78 ms; *n* = 7; *p* < 0.01; **Figure 8B**), and induced the hyperpolarization of membrane potential (pre: -54.96 ± 1.81 mV; carbachol + mecamylamine: -65.63 ± 2.25 mV; wash: -56.76 ± 2.28 mV; *n* = 7; *p* < 0.01; **Figure 8C**). These results suggested that mecamylamine failed to inhibit the effects of carbachol on RA PNs.

### Effects of nAChR Antagonist on the Intrinsic Properties of RA PNs

**FIGURE 9:**
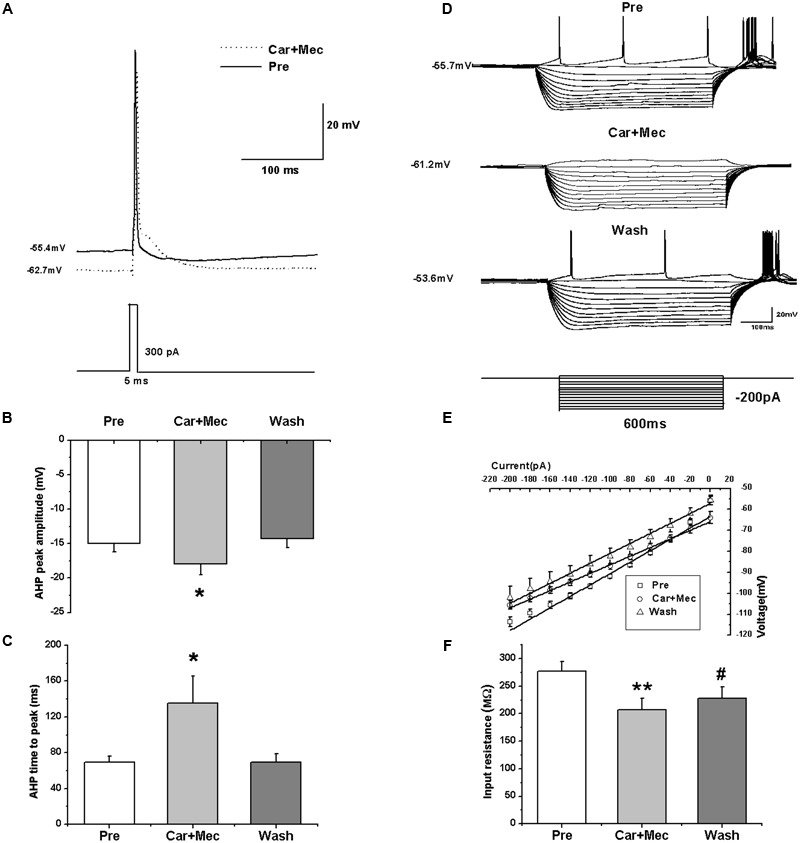
**Effects of carbachol (Car, 30 μM) + mecamylamine (Mec, 10 μM) on the intrinsic properties of RA PNs. (A)** Representative AP recordings in response to a depolarizing pulse of 300 pA at 5 ms duration before and during Car + Mec application. **(B)** AHP peak amplitude was significantly increased in the presence of Car + Mec (*n* = 7). **(C)** AHP time to peak was significantly increased in the presence of Car + Mec (*n* = 7). **(D)** Voltage responses of a neuron to a series of hyperpolarizing current steps before, during and after Car + Mec application. **(E)** The current-voltage curves showed a significant change in slope during Car + Mec application (*n* = 8), indicating Car + Mec had an effect on the membrane input resistance. **(F)** Membrane input resistance was significantly decreased in the presence of Car + Mec (*n* = 8). ^∗∗^*p* < 0.01, ^∗^*p* < 0.05, #*p* < 0.05 vs. Pre.

We further analyzed the effects of carbachol plus mecamylamine on the intrinsic properties of RA PNs (**Figure 9A**). Similar to the effects of carbachol alone, the AHP peak amplitude and AHP time to peak were increased during the application of carbachol plus mecamylamine, and returned to the control level after carbachol plus mecamylamine washout (**Table 2** and **Figures 9B,C**), along with significant hyperpolarization of the membrane potential and gradual recovery (**Table 2**). However, the AP threshold, peak amplitude and half-width were unaffected (**Table 2**). In addition, membrane input resistance, membrane time constant and membrane capacitance were significantly decreased following application of carbachol and mecamylamine, and restored eventually after the washout (**Table 2** and **Figures 9D–F**). These results further suggested that nAChR antagonist failed to inhibit the effects of carbachol on RA PNs, indicating that nAChRs were not involved in the cholinergic modulation of the intrinsic properties of RA PNs.

**Table 2:** Measurements of intrinsic properties of RA PNs before, during and after carbachol (Car, 30 μM) + mecamylamine (Mec, 10 μM) application.

Parameters	Pre	Car + Mec	*t* values, *p* values	Wash
Membrane potential (mV, *n* = 7)	-57.17 ± 2.12	-63.36 ± 2.99*	*t* = 3.016, *p* = 0.024	-57.95 ± 2.32
AP threshold (mV, *n* = 7)	-44.19 ± 3.86	-44.49 ± 3.95	*t* = -0.287, *p* = 0.784	-44.79 ± 3.44
Peak amplitude (mV, *n* = 7)	65.15 ± 3.90	64.65 ± 6.36	*t* = 0.117, *p* = 0.915	71.75 ± 3.96
Half-width (ms, *n* = 7)	2.03 ± 0.16	1.93 ± 0.16	*t* = 2.291, *p* = 0.062	1.96 ± 0.18
AHP peak amplitude (mV, *n* = 7)	-15.00 ± 1.31	-17.97 ± 1.66*	*t* = 3.548, *p* = 0.012	-14.28 ± 1.44
AHP time to peak (ms, *n* = 7)	69.11 ± 7.73	135.23 ± 32.64*	*t* = -2.479, *p* = 0.048	69.27 ± 10.13
Membrane input resistance (MΩ, *n* = 8)	277.14 ± 19.21	207.25 ± 21.56**	*t* = 4.823, *p* = 0.002	227.55 ± 22.85#
Membrane time constant (ms, *n* = 8)	25.69 ± 2.88	17.31 ± 2.33**	*t* = 5.411, *p* = 9.960E-4	19.58 ± 2.19
Membrane capacitance (pF, *n* = 8)	94.05 ± 10.39	84.07 ± 9.27*	*t* = 2.398, *p* = 0.048	89.37 ± 9.74

*^∗^*p <* 0.05, ^∗∗^*p <* 0.01, #*p* < 0.05 vs. Pre.*

## Discussion

In the present study, we observed strong effects of cholinergic agents on the PNs in the song premotor nucleus RA of adult male zebra finches. We found that the effects of the cholinergic analog carbachol on RA PNs were mimicked by the mAChR agonist muscarine, which significantly decreased the evoked AP firing frequency, along with hyperpolarization of the membrane potential, increase in the evoked AP latency, AHP peak amplitude and AHP time to peak, and decrease in the membrane input resistance and membrane time constant. The AP threshold, peak amplitude, half-width and membrane capacitance were not affected by muscarine. Furthermore, the mAChR antagonist atropine blocked the effects of carbachol. In addition, the activity of RA PNs was not affected by the nAChR agonist nicotine or DMPP. However, the nAChR antagonist mecamylamine failed to block the effects of carbachol. These results indicated that the effects of carbachol on the intrinsic properties of RA PNs were mediated mainly via activation of mAChRs but not nAChRs.

Songbird RA PNs are similar to pyramidal tract neurons in the lower layer 5 of mammalian motor cortex (Pfenning et al., 2014). ACh plays an important role in the regulation of mammalian cortex activity (Kalmbach and Waters, 2014). In mammals, local cholinergic activation within the motor cortex modulates cortical map plasticity and motor learning (Conner et al., 2010). Hess and Krawczyk (1996) showed that application of carbachol resulted in reduction of field potentials evoked in layer 2/3 horizontal connections of rat motor cortex via M1 mAChRs. A previous study by Mittmann and Alzheimer (1998) showed that carbachol decreased the persistent Na^+^ current in rat neocortical pyramidal neurons via mAChRs. In addition, the previous study employing cell-attached recording by Yang et al. (2010) demonstrated that carbachol inhibited excitability of rat suprachiasmatic nucleus neurons. The effect was mimicked by mAChR agonists, but not nAChR agonists. It was reported that multiple types of response to carbachol in rat parafascicular neurons were mainly mediated via direct activation of post-synaptic mAChRs (Ye et al., 2009).

An early quantitative autoradiographic study showed that either high or low levels of mAChR labels were detected in song control nuclei of songbirds, including nucleus RA (Ball et al., 1990). Perhaps, the cholinergic modulation of RA PNs is “direct,” and mediated by post-synaptic mAChRs on RA PNs. Further studies suggested that presynaptic ACh receptors may also mediate the regulation of synaptic transmission and neuronal activity (Gigout et al., 2012). For example, in the basolateral amygdaloid pyramidal-type neurons of rats, carbachol rapidly excited presynaptic GABAergic interneurons by binding to mAChRs, and depressed the excitability of post-synaptic neurons (Washburn and Moises, 1992). GABAergic interneurons occur abundantly in the song premotor nucleus RA of songbirds. It is also possible that carbachol indirectly reduced the excitability of RA PNs via activation of mAChRs on the presynaptic GABAergic interneurons. Interestingly, Zhang and Warren (2002) reported that ACh or carbachol affect presynaptic mAChRs and nAChRs, respectively, in rat nucleus accumbens (nAcb) neurons during postnatal development, and decrease or increase glutamatergic neurotransmission. Finally, the excitatory effect of ACh or carbachol mediated by nAChRs was masked by the inhibitory effect of mAChRs. The synaptic components of RA are similar to those of rat nAcb in terms of glutamatergic synaptic inputs received from HVC and LMAN (Sizemore and Perkel, 2008). Thus, these studies provide multiple possible explanations for our results. A further study is needed to validate these findings.

The study of Salgado-Commissariat et al. (2004) showed that nicotine increases the excitability of RA neurons, which is inconsistent with our findings. RA includes two types of neurons: PNs and interneurons, and the previous study does not elucidate the type of neuron investigated. In our study, the results showed that the two types of nAChR agonists, including nicotine and DMPP, had no effect on the excitability of RA PNs. Co-application of carbachol and atropine, which is supposed to activate nAChRs alone, also had no effect on the excitability of RA PNs. Furthermore, the Mello lab zebra finch *in situ* hybridization database suggests the expression of muscarinic receptors but not nicotinic receptors in RA (Lovell et al., 2008), which supports our findings.

Robust nucleus of the arcopallium is a key nucleus of songbirds involved in the regulation of song behavior. PNs occurring in the premotor nucleus RA project to vocal and respiratory control nuclei in the midbrain and brainstem. The activity of RA PNs generates precise neural signals to drive song production (Chi and Margoliash, 2001). Our results, acquired from the brain slices of adult male zebra finch, demonstrate that cholinergic neurotransmitters modulate the activity of RA PNs by affecting intrinsic membrane properties via activation of mAChRs. Intrinsic membrane properties play a major role in the regulation of neuronal behavior (Surges et al., 2004). Thus, our studies provide *in vitro* electrophysiological evidence supporting the cholinergic modulation of RA PNs, and also provide a cellular mechanism underlying the cholinergic regulation of song behavior. It suggests that endogenous ACh regulates song production by affecting intrinsic membrane properties of RA PNs via mAChRs under physiological conditions. The study of Shea and Margoliash (2003) shows that injections of carbachol or muscarine into HVC of anesthetized zebra finches significantly altered the discharge rates and auditory responsiveness in both HVC and RA, and nicotine produced similar effects in HVC. Shea et al. (2010) further provided *in vitro* electrophysiological evidence supporting the cholinergic modulation of HVC neurons using whole cell recordings. Therefore, under physiological conditions, ACh affects song behavior by modulating the activities of song premotor nuclei. The cholinergic system, arising from the basal forebrain of songbirds, is involved in the regulation of song motor control and song learning. These findings contribute to the understanding of mechanisms associated with cholinergic regulation in birdsong production.

## Author Contributions

WM and SW contributed equally to this study. WM and SW designed and performed the experiments, analyzed the data and wrote this manuscript. LY and NZ were involved in analyzing a portion of the data. DL contributed reagents, materials and analytical tools.

## Conflict of Interest Statement

The authors declare that the research was conducted in the absence of any commercial or financial relationships that could be construed as a potential conflict of interest.

## References

[B1] Ball G. F. Nock B. Wingfield J. C. McEwen B. S. Balthazart J. (1990). Muscarinic cholinergic receptors in the songbird and quail brain: a quantitative autoradiographic study. *J. Comp. Neurol.* 298 431–442. 10.1002/cne.902980405 2229474

[B2] Brainard M. S. Doupe A. J. (2002). What songbirds teach us about learning. *Nature* 417 351–358. 10.1038/417351a 12015616

[B3] Chi Z. Margoliash D. (2001). Temporal precision and temporal drift in brain and behavior of zebra finch song. *Neuron* 32 899–910. 10.1016/S0896-6273(01)00524-4 11738034

[B4] Conner J. M. Kulczycki M. Tuszynski M. H. (2010). Unique contributions of distinct cholinergic projections to motor cortical plasticity and learning. *Cereb. Cortex* 20 2739–2748. 10.1093/cercor/bhq022 20181623 PMC2951849

[B5] Farries M. A. Perkel D. J. (2000). Electrophysiological properties of avian basal ganglia neurons recorded in vitro. *J. Neurophysiol.* 84 2502–2513. 11067993 10.1152/jn.2000.84.5.2502

[B6] Foster E. F. Bottjer S. W. (2001). Lesions of a telencephalic nucleus in male zebra finches: influences on vocal behavior in juveniles and adults. *J. Neurobiol.* 46 142–165. 10.1002/1097-4695(20010205)46:2<142::AID-NEU60>3.0.CO;2-R 11153015

[B7] Gigout S. Jones G. A. Wierschke S. Davies C. H. Watson J. M. Deisz R. A. (2012). Distinct muscarinic acetylcholine receptor subtypes mediate pre- and postsynaptic effects in rat neocortex. *BMC Neurosci.* 13:42 10.1186/1471-2202-13-42 PMC3416661 22540185

[B8] Hess G. Krawczyk R. (1996). Cholinergic modulation of synaptic transmission in horizontal connections of rat motor cortex. *Acta Neurobiol. Exp. (Wars)* 56 863–872. 9033122 10.55782/ane-1996-1193

[B9] Jarvis E. D. (2004). Learned birdsong and the neurobiology of human language. *Ann. N. Y. Acad. Sci.* 1016 749–777. 10.1196/annals.1298.0381016/1/749 15313804 PMC2485240

[B10] Kalmbach A. Waters J. (2014). Modulation of high- and low-frequency components of the cortical local field potential via nicotinic andmuscarinic acetylcholine receptors in anesthetized mice. *J. Neurophysiol.* 111 258–272. 10.1152/jn.00244.2013 24155009 PMC3921379

[B11] Li R. Zuo M. X. Sakaguchi H. (1999). Auditory-vocal cholinergic pathway in zebra finch brain. *Neuroreport* 10 165–169. 10.1097/00001756-199901180-00032 10094156

[B12] Liao S. Q. Hou G. Q. Liu X. L. Long C. Li D. F. (2011). Electrophysiological properties of neurons in the robust nucleus of the arcopallium of adult male zebra finches. *Neurosci. Lett.* 487 234–239. 10.1016/j.neulet.2010.10.029 20969922

[B13] Lovell P. V. Clayton D. F. Replogle K. L. Mello C. V. (2008). Birdsong “transcriptomics”: neurochemical specializations of the oscine song system. *PLoS ONE* 3:e3440 10.1371/journal.pone.0003440 PMC2563692 18941504

[B14] Meitzen J. Weaver A. L. Brenowitz E. A. Perkel D. J. (2009). Plastic and stable electrophysiological properties of adult avian forebrain song-control neurons across changing breeding conditions. *J. Neurosci.* 29 6558–6567. 10.1523/JNEUROSCI.5571-08.2009 19458226 PMC2722045

[B15] Meng W. Wang S. H. Li D. F. (2016). Carbachol-induced reduction in the activity of adult male zebra finch RA projection neurons. *Neural Plast.* 2016:7246827 10.1155/2016/7246827 PMC4745321 26904300

[B16] Mittmann T. Alzheimer C. (1998). Muscarinic inhibition of persistent Na+ current in rat neocortical pyramidal neurons. *J. Neurophysiol.* 79 1579–1582. 9497434 10.1152/jn.1998.79.3.1579

[B17] Nottebohm F. Stokes T. M. Leonard C. M. (1976). Central control of song in the canary, *Serinus canarius*. *J. Comp. Neurol.* 165 457–486. 10.1002/cne.901650405 1262540

[B18] Pfenning A. R. Hara E. Whitney O. Rivas M. V. Wang R. Roulhac P. L. (2014). Convergent transcriptional specializations in the brains of humans and song-learning birds. *Science* 346:1256846 10.1126/science.1256846 PMC4385736 25504733

[B19] Rouse M. L. Jr. Ball G. F. (2016). Lesions targeted to the anterior forebrain disrupt vocal variability associated with testosterone-induced sensorimotor song development in adult female canaries, *Serinus canaria*. *Dev. Neurobiol.* 76 3–18. 10.1002/dneu.22295 25864444 PMC4600635

[B20] Ryan S. M. Arnold A. P. (1981). Evidence for cholinergic participation in the control of bird song; acetylcholinesterase distribution and muscarinic receptor autoradiography in the zebra finch brain. *J. Comp. Neurol.* 202 211–219. 10.1002/cne.902020207 7298898

[B21] Sadananda M. (2004). Acetylcholinesterase in central vocal control nuclei of the zebra finch (*Taeniopygia guttata*). *J. Biosci.* 29 189–200. 10.1007/BF02703417 15286416

[B22] Salgado-Commissariat D. Rosenfield D. B. Helekar S. A. (2004). Nicotine-mediated plasticity in robust nucleus of the archistriatum of the adult zebra finch. *Brain Res.* 1018 97–105. 10.1016/j.brainres.2004.05.051 15262210

[B23] Shea S. D. Koch H. Baleckaitis D. Ramirez J. M. Margoliash D. (2010). Neuron-specific cholinergic modulation of a forebrain song control nucleus. *J. Neurophysiol.* 103 733–745. 10.1152/jn.00803.2009 19939956 PMC2822690

[B24] Shea S. D. Margoliash D. (2003). Basal forebrain cholinergic modulation of auditory activity in the zebra finch song system. *Neuron* 40 1213–1226. 10.1016/S0896-6273(03)00723-2 14687554

[B25] Sizemore M. Perkel D. J. (2008). Noradrenergic and GABA B receptor activation differentially modulate inputs to the premotor nucleus RA in zebra finches. *J. Neurophysiol.* 100 8–18. 10.1152/jn.01212.2007 18463188 PMC4071943

[B26] Sober S. J. Wohlgemuth M. J. Brainard M. S. (2008). Central contributions to acoustic variation in birdsong. *J. Neurosci.* 28 10370–10379. 10.1523/JNEUROSCI.2448-2408.2008 18842896 PMC2613831

[B27] Spiro J. E. Dalva M. B. Mooney R. (1999). Long-range inhibition within the zebra finch song nucleus RA can coordinate the firing of multiple projection neurons. *J. Neurophysiol.* 81 3007–3020. 10368416 10.1152/jn.1999.81.6.3007

[B28] Surges R. Freiman T. M. Feuerstein T. J. (2004). Input resistance is voltage dependent due to activation of Ih channels in rat CA1 pyramidal cells. *J. Neurosci. Res.* 76 475–480. 10.1002/jnr.20075 15114619

[B29] Vicario D. S. (1994). Motor mechanisms relevant to auditory-vocal interactions in songbirds. *Brain Behav. Evol.* 44 265–278. 10.1159/000113581 7842285

[B30] Wang S. Liao C. Li F. Liu S. Meng W. Li D. (2014). Castration modulates singing patterns and electrophysiological properties of RA projection neurons in adult male zebra finches. *PeerJ* 2:e352 10.7717/peerj.352 PMC3994634 24765586

[B31] Wang S. Liao C. Meng W. Huang Q. Li D. (2015). Activation of D1-like dopamine receptors increases the NMDA-induced gain modulation through a PKA-dependent pathway in the premotor nucleus of adult zebra finches. *Neurosci. Lett.* 589 37–41. 10.1016/j.neulet.2015.01.032 25596438

[B32] Washburn M. S. Moises H. C. (1992). Muscarinic responses of rat basolateral amygdaloid neurons recorded in vitro. *J. Physiol.* 449 121–154. 10.1113/jphysiol.1992.sp019078 1522506 PMC1176071

[B33] Watson J. T. Adkins-Regan E. Whiting P. Lindstrom J. M. Podleski T. R. (1988). Autoradiographic localization of nicotinic acetylcholine receptors in the brain of the zebra finch (*Poephila guttata*). *J. Comp. Neurol.* 274 255–264. 10.1002/cne.902740209 3209741

[B34] Yang J. J. Wang Y. T. Cheng P. C. Kuo Y. J. Huang R. C. (2010). Cholinergic modulation of neuronal excitability in the rat suprachiasmatic nucleus. *J. Neurophysiol.* 103 1397–1409. 10.1152/jn.00877.2009 20071625

[B35] Yao L. H. Li C. H. Yan W. W. Huang J. N. Liu W. X. Xiao P. (2011). Cordycepin decreases activity of hippocampal CA1 pyramidal neuron through membrane hyperpolarization. *Neurosci. Lett.* 503 256–260. 10.1016/j.neulet.2011.08.048 21896311

[B36] Ye M. Hayar A. Garcia-Rill E. (2009). Cholinergic responses and intrinsic membrane properties of developing thalamic parafascicular neurons. *J. Neurophysiol.* 102 774–785. 10.1152/jn.91132.2008 19474169 PMC2724366

[B37] Yu A. C. Margoliash D. (1996). Temporal hierarchical control of singing in birds. *Science* 273 1871–1875. 10.1126/science.273.5283.1871 8791594

[B38] Zhang L. Warren R. A. (2002). Muscarinic and nicotinic presynaptic modulation of EPSCs in the nucleus accumbens during postnatal development. *J. Neurophysiol.* 88 3315–3330. 10.1152/jn.01025.2001 12466449

